# Trait dissociation is associated with dissociative experiences arising from disrupted multisensory integration

**DOI:** 10.1038/s41598-025-97320-9

**Published:** 2025-04-12

**Authors:** Jamie A. Moffatt, Marte Roel Lesur, Bigna Lenggenhager, Marieke L. Weijs, Valerio Maglianella, Hugo D. Critchley, Sarah N. Garfinkel, Kathryn Greenwood

**Affiliations:** 1 School of Psychology, University of Sussex, Falmer, UK; 2 Department of Psychology, Royal Holloway, University of London, London, UK; 3 Department of Psychology, University of Zurich, Zurich, Switzerland; 4 Department of Informatics, Universidad Carlos III de Madrid, Madrid, Spain; 5 Department of Psychology, University of Konstanz, Konstanz, Germany; 6 Department of Health Sciences and Technology, ETH Zurich, Zurich, Switzerland; 7 Brighton and Sussex Medical School, Falmer, UK; 8 Sussex Partnership NHS Foundation Trust, Brighton, UK; 9 Institute of Cognitive Neuroscience, University College London, London, UK

**Keywords:** Dissociation, Multi-sensory integration, Mixed reality, Embodiment, Interoception, Self-action, Psychology, Human behaviour

## Abstract

Dissociation is a transdiagnostic mental health symptom involving a sense of detachment from one’s own body. A coherent percept of our body relies upon the smooth integration of different senses, such as vision and touch, which are processed by the brain at different speeds. We investigated the association between multi-sensory integration and dissociation in a *N* = 100 non-clinical sample. An immersive head-mounted display provided participants with a first-person view of their body while their arm was brushed by themselves or by the researcher. This mixed-reality setup allowed for the systematic variation of a delay to the visual feed, resulting in visual information being delayed relative to other senses (e.g. touch). Larger delays were associated with greater self-reported feelings of dis-ownership from the body. In addition, participants with high trait dissociation felt elevated sensations of dis-ownership from the body and were more sensitive to the multi-sensory delay, suggesting an increased tendency to ‘fraction’ senses in dissociation. Moreover, individuals with higher dissociation displayed similar cardiac reactivity to both self and other touch. These findings highlight two key mechanisms underlying dissociation; altered sensory-integration and increased awareness of self-action.

**Supplementary Information:**

The online version contains supplementary material available at 10.1038/s41598-025-97320-9.

## Introduction

The sense of body ownership describes the feeling that a whole body, or a body part, is experienced as belonging to oneself. Although this is typically intact for most people, those with dissociative experiences often describe feeling a confusing and distressing loss of ownership over their own body, alongside other experiences such as feeling detached from the world, from one’s emotions or from the sense of self^1,2^. Dissociative experiences form the core symptoms of dissociative disorders such as depersonalization-derealization disorder, but also occur in the general population^3^ and are transdiagnostic, occurring in PTSD, borderline personality disorder, eating disorders, anxiety, depression and schizophrenia^4,5^. Dissociation may play a key role in the development of other types of anomalous experiences. For example, individuals deemed to be at risk of developing psychosis are more likely to transition to a full psychiatric diagnosis if they also report dissociation^6^. In addition, network-^7^ and meta-analyses^8^ show robust associations between dissociation and other aberrant experiences including hallucinations, delusions and paranoia in clinical and non-clinical populations. It is therefore vital to understand how dissociative experiences, exemplified by a loss of ownership over the body, can occur.

A coherent perception of the body relies upon the ability to accurately integrate sensations from multiple sources, with sensory information about the body arising from exteroceptive (e.g. vision, touch), interoceptive (e.g. feelings of hunger) and proprioceptive (e.g. posture) sources. Sensory inputs from different modalities are processed by the brain at varying speeds. As an illustrative example, new information arising in the visual field reaches the retina and is subsequently processed quicker than somatosensory inputs, which must ascend from tactile receptors in the skin through the central nervous system to the brain. Slower still are interoceptive sensory inputs, where novel interoceptive signals such as an accelerated heart rate or a sharp intake of breath are processed over multiple seconds^9^. Despite these naturally occurring delays between sensations, the brain is typically successful in combining them to form coherent percept of the body^10^ and the body in the world. Causal inference accounts of multi-sensory integration state that two senses are integrated together if the observer infers that they have arisen from the same source, with greater temporal and spatial discrepancies between senses making causal inference (and therefore integration) less likely^11^. The sense of the body is thought to arise from the same principles that govern multi-sensory integration^12–14^, which is supported by a wide range of studies demonstrating how manipulation of temporal and spatial timings of multi-sensory stimuli can result in altered senses of the body^15^. A classic example is the Rubber Hand Illusion (RHI), where viewing a fake rubber hand being touched while simultaneously feeling the touch to one’s own real hand (and thus experiencing an artificially induced multi-sensory pairing of seen and felt touch) can induce a sense of body ownership over the rubber hand^16–18^. Although the RHI demonstrates how the sense of body ownership relies upon the integration of senses, unusual bodily experiences that occur in dissociation more frequently involve a sense of detachment rather than an extension of the body^2,19^.

Mixed-reality approaches to experimentally disrupt multi-sensory integration can reduce the sense of body ownership over one’s own body^20–22^. The mixed-reality approach uses a webcam attached to a head-mounted display to provide a first-person perspective of one’s own body and surroundings in real time, while allowing for digital manipulation of the visual feed. Delaying the visual feed during a brushstroke to the arm, thereby separating out the visual and tactile sensation of a touch, reduces feelings of body ownership compared to when no delay is applied in a non-clinical sample^20^. Reduced body ownership was also found to accompany delayed visual perception of touch that was self-initiated, though to a lesser extent than other-initiated touch^21^. These induced experiences of body dis-ownership appear to more closely resemble experiences of trait dissociation than the experience of extended embodiment triggered by the RHI. However, the association between dissociation and induced experiences of a loss of body ownership has yet to be directly tested.

The mixed-reality approach for experimentally inducing a loss of ownership over the body provides an opportunity to examine additional mechanisms of dissociation. As feelings of body ownership are proposed to arise from the integration of senses from different modalities, individual differences in unisensory mechanisms may also influence susceptibility to experimentally induced dissociative experiences. Indeed, individuals better at proprioceptive judgements are less prone to the RHI^23^. In a similar fashion, greater accuracy in perceiving tactile sensations delivered to the body during the mixed-reality task may protect against disrupted feelings of body ownership.

Beyond the exteroceptive and proprioceptive domains, the sense of body ownership is also informed by internally generated sensations^24–26^. Interoception refers to the sensing of internal bodily stimuli, including heartbeats or feelings of thirst and hunger^27^, and can be assessed at several levels ranging from unconscious biological processes, such as the quality of afferent signals transmitted from the body to the brain, to the accuracy of consciously perceived heartbeats and other internal sensations^28^. Disrupted interoceptive processing and brain-body connectivity appears to be linked to dissociation^29^. Typically, startle responses to intrusive auditory stimuli are attenuated if the sound is delivered at phases of the cardiac cycle coinciding with heart-brain communication^30^. However, patients with a dissociative disorder displayed no such attenuation, suggesting weakened afferent cardiac signals to the brain^31^. Additionally, people who experience dissociation tend to have reduced accuracy in consciously tracking their own heartbeats^32,33^. Reduced interoceptive ability is also associated with stronger body ownership effects on the RHI^26^, suggesting that there may be an association between altered body-brain connectivity, defined as reduced interoceptive ability, and susceptibility to experimentally induced body ownership and dissociative experiences.

Finally, whilst findings from studies using the RHI and mixed-reality highlight the importance of ‘bottom-up’ multi-sensory integration processes in defining the sense of the body, there is also growing evidence of how ‘top-down’ predictions (priors or ‘beliefs’) contribute to the sense of body ownership. Arising from research on hypnotic suggestion, ‘phenomenological control’ refers to the ability to adapt perception according to a suggestion or a belief^34^ and can be assessed with validated paradigms testing how strongly individuals respond to suggestions^35^. Those with greater phenomenological control also show greater embodiment over the fake hand on the RHI^32^, though see^33,34^, implying that the extension of the body is at least partly accounted for by a willingness to match experience with suggestion. It is currently unclear how phenomenological control may relate to a loss of ownership over the body.

**Fig. 1:**
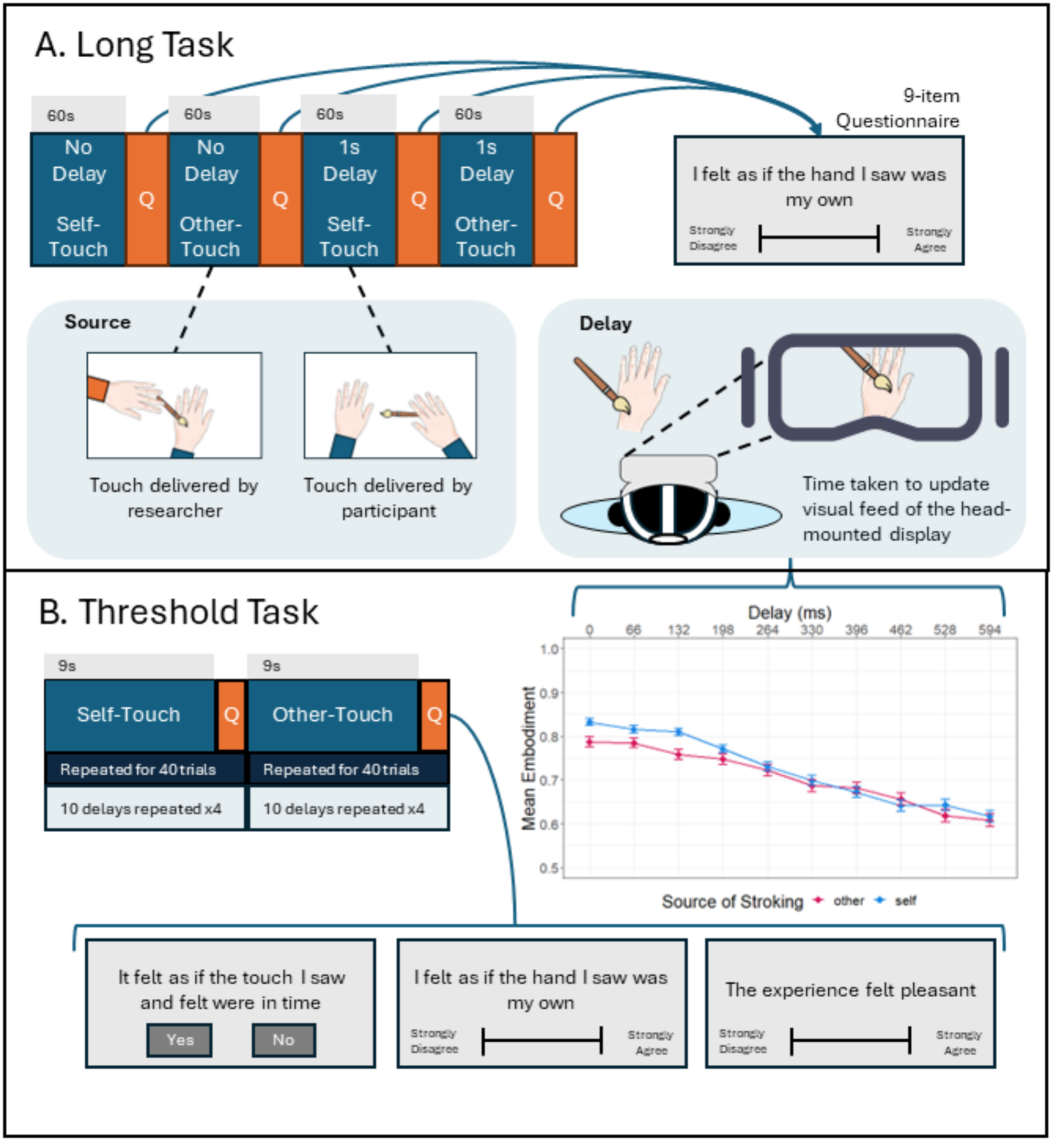
Mixed-Reality Task Procedures. (**A**) In the ‘Long’ task, participants viewed their arm being brushed by themselves (self-touch) or by the researcher (other-touch) for 60s whilst the visual feed of the head-mounted display was delayed by either 0–1 s, plus the intrinsic delay of the system (Mean = 134.23ms, see supplementary materials). The order of the four different trial types were counterbalanced across participants. After each trial, participants completed a 9-item questionnaire rating their feelings of dis-ownership, derealization, de-afference, embodiment and pleasantness (see Table 4 for full questionnaire). (**B**) The ‘Threshold’ task involved 40 self-touch and 40 other-touch trials, the order of which was counterbalanced. Each trial lasted 9s and the delay ranged from 0-594ms. After each trial, participants judged the synchrony of the seen and felt touch and rated their felt embodiment and pleasantness on visual analogue scales. Self-reported embodiment decreased monotonically with increasing delay (*N* = 90), points indicate mean embodiment ratings, error bars depict standard error from the mean.

The present study employed a mixed-reality approach to separate out normally integrated visuo-tactile sensations^20^ in a non-clinical population. A “long” version of the mixed-reality task assessed reported feelings of body dis-ownership after longer periods (60s) of a fixed 1 s delay or no delay to visual perception of a brushstroke to the hand. Additionally, a “threshold” version employed multiple delay times to assess more comprehensively the relation between the delay and feelings of body ownership. Both versions included self- and other-stroking conditions in within-participants designs (see Fig. 1). In both tasks we expected self-reported feelings of dis-ownership from the body to be greater in conditions with a delay between vision and touch compared to no delay, and in conditions with other-touch compared to self-touch. Selected questionnaires assessed trait dissociative and anomalous perceptual experiences, and we expected heightened trait scores to be associated with heightened feelings of body dis-ownership in response to the mixed-reality paradigms. Interoceptive ability was assessed with a heartbeat discrimination task^39,40^, tactile acuity was measured with a grating discrimination task^41^ and phenomenological control was assessed with a shortened version of the Sussex-Waterloo Hypnotisability Scale (SWASH^35^). We expected experimentally induced feelings of body dis-ownership to be negatively associated with interoceptive and tactile perceptual abilities, but positively associated with phenomenological control scores. Finally, physiological responses associated with experimentally induced dissociative experiences were examined in a subsample of participants in whom heart data were recorded during the mixed-reality task. The study protocol, key hypotheses, variables of interest and analysis plan were pre-registered (https://osf.io/qh543/), after the end of data collection but prior to analysis of the data.

## Results

### Sample characteristics

100 participants without current or historic diagnosis of a psychiatric, biological or neurological disorder were recruited to take part in the study. Participants were mostly young, female, right-handed and well-educated, but encompassed a diverse set of ethnic backgrounds (Table 1). Some participants were unable to complete some parts of the study, so counts of missing or excluded data for each task and reasons for missingness are detailed in the supplementary materials.

**Table 1:** Demographics table.

Variable	Value
Age in years, mean (SD)	22.37 (3.59)
Years of Education, mean (SD)	15.56 (2.53)
Gender	
Female (%)	77
Male (%)	22
Transgender (%)	1
Ethnicity	
White (%)	46
Mixed/Multiple Ethnic Groups (%)	10
Asian/Asian British (%)	27
Black/African/Caribbean/Black British (%)	7
Other (%)	7
Prefer Not to Say (%)	3
Alcohol Use (Last 12 Months)	
Never (%)	17
Monthly or Less (%)	30
Two to Four Times a Month (%)	29
Two or Three Times a Week (%)	22
More than Four Times a Week (%)	2
Cannabis Use (Last 12 Months)	
Never (%)	74
Monthly or Less (%)	22
Two to Four Times a Month (%)	1
Two or Three Times a Week (%)	3
More than Four Times a Week (%)	0
Handedness	
Right-Handed (%)	94
Left-Handed (%)	3
Mixed (%)	3

*n* = 100. SD = Standard Deviation.

### Influence of multi-sensory disruption on body ownership

First we tested whether participants felt a sense of dis-ownership from their body in response to a fixed 1s delay between visual and tactile sensations of brushstrokes to the arm over a long (60s) period (see Fig. 1A). A linear mixed model (*N* = 87), with Delay (No Delay, 1s Delay), Source of Stroking (Self, Other) and the interaction between two included as fixed effects was specified, with random slopes included for Delay, Source and the interaction term, as well as a by-subject random intercept. The model found that dis-ownership ratings were significantly greater when there was a delay, $\:\widehat{\beta\:}=-0.33$, 95% CI $\:\left[-0.38,-0.28\right]$, $\:t\left(86.00\right)=-12.79$, $\:p<0.001$. Neither source of stroking, $\:\widehat{\beta\:}=-0.02$, 95% CI $\:\left[-0.05,0.00\right]$, $\:t\left(86.00\right)=-1.78$, $\:p=0.078$, nor the interaction between Delay and Source, $\:\widehat{\beta\:}=-0.03$, 95% CI $\:\left[-0.09,0.04\right]$, $\:t\left(86.00\right)=-0.79$, $\:p=0.431$ were significant.

Similar linear mixed models were conducted to determine if source and delay significantly influenced ratings of derealisation, deafference, embodiment and pleasantness. Compared to no delay conditions, a 1s delay led to significantly greater ratings of derealization, $\:\widehat{\beta\:}=-0.24$, 95% CI $\:\left[-0.29,-0.20\right]$, $\:t\left(85.99\right)=-10.80$, $\:p<0.001$, and deafference, $\:\widehat{\beta\:}=-0.22$, 95% CI $\:\left[-0.26,-0.18\right]$, $\:t\left(86.00\right)=-10.32$, $\:p<0.001$, but significantly reduced ratings of embodiment, $\:\widehat{\beta\:}=0.19$, 95% CI $\:\left[0.14,0.24\right]$, $\:t\left(86.00\right)=7.51$, $\:p<0.001,$ and pleasantness, $\:\widehat{\beta\:}=0.12$, 95% CI $\:\left[0.07,0.17\right]$, $\:t\left(86.00\right)=5.02$, $\:p<0.001$. Other-touch led to significantly greater ratings of derealisation, $\:\widehat{\beta\:}=-0.03$, 95% CI $\:\left[-0.06,0.00\right]$, $\:t\left(86.00\right)=-2.10$, $\:p=0.038$ but reduced embodiment, $\:\widehat{\beta\:}=0.03$, 95% CI $\:\left[0.00,0.05\right]$, $\:t\left(86.00\right)=2.24$, $\:p=0.028$, compared to self-touch. None of the interactions between source and delay were significant except for pleasantness ratings, $\:\widehat{\beta\:}=0.05$, 95% CI $\:\left[0.01,0.10\right]$, $\:t\left(86.00\right)=2.26$, $\:p=0.026$. Post-hoc t-tests to explore this interaction found that pleasantness ratings were significantly higher for the No Delay compared to Delay condition for both self-touch, $\:t\:\left(86\right)=-5.63,\:p<0.001$, and other-touch, $\:t\:\left(86\right)=-3.47,\:p<0.001$. However, pleasantness was not significantly different between self-touch and other-touch at either Delay, $\:t\:\left(86\right)=1.60,\:=0.114$, or at No Delay, $\:t\:\left(86\right)-1.35,\:p=0.180$. The significant interaction therefore likely arose from a cross-over effect, whereby pleasantness was (non-significantly) higher for self-touch compared to other-touch in trials with no delay, but (non-significantly) higher for other-touch compared to self-touch when there was a delay

**Fig. 2:**
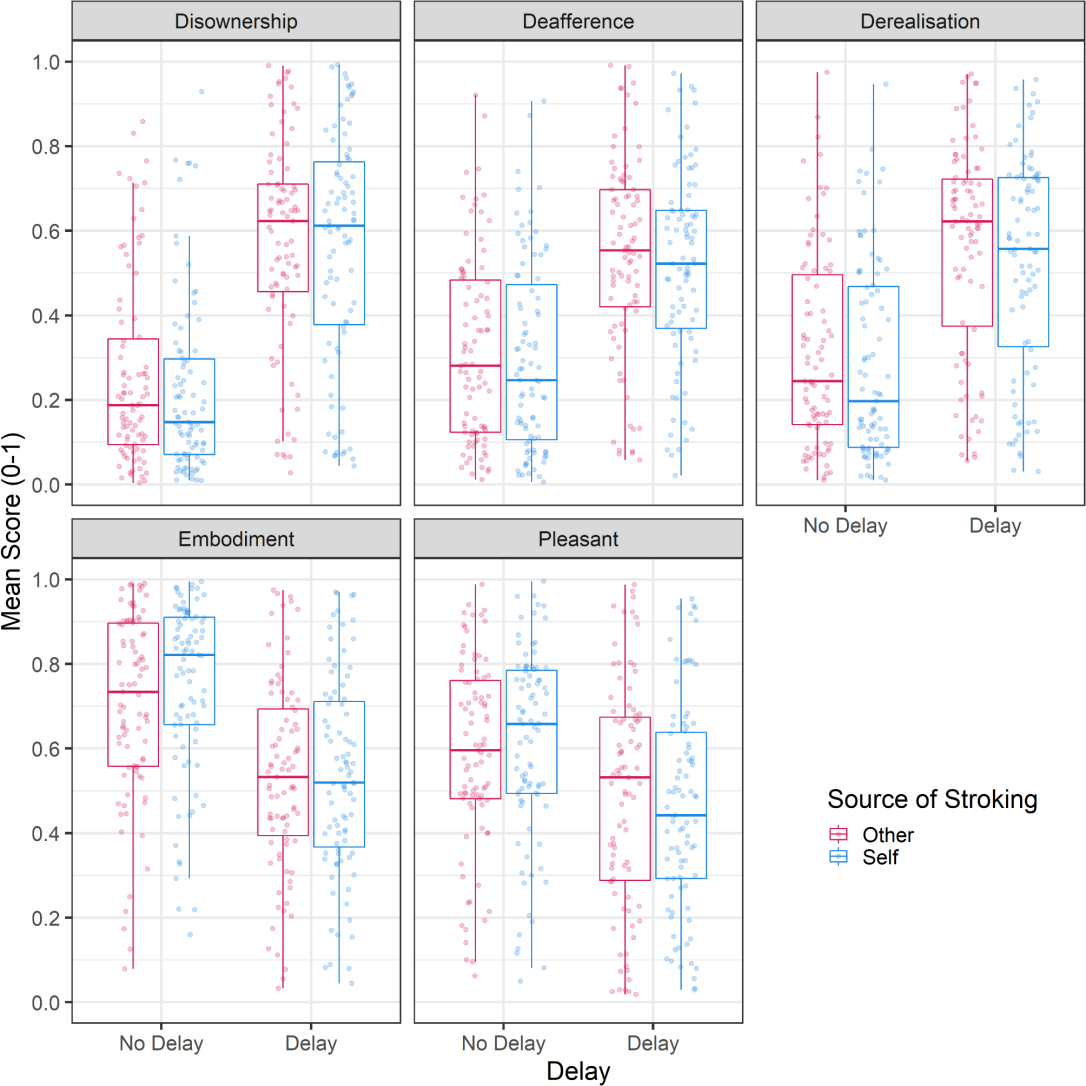
Tukey boxplots displaying average ratings of disownership, deafference, derealisation, embodiment and pleasantness. Delay refers to a one-second delay applied to the visual feed of the head-mounted display whilst a brushstroke was delivered to the arm, in addition to the intrinsic delay of the system. Source of stroking describes whether the brushstroke was delivered by the researcher (Other) or the participant themselves (Self). Points are individual ratings, jittered horizontally. The central line refers to the median values, and the lower and upper hinges refer to the 1st and 3rd percentiles respectively. The lower and upper whiskers extend to 1.5 times the inter-quartile range (distance between 1st and 3rd percentiles). *N* = 87.

Together these results suggest that a 1s time delay between seen and felt sensations of touch increased feelings of dis-ownership, derealisation and de-afference, and reduced feelings of embodiment and pleasantness (see Fig. 2). In addition, self-touch led to reduced derealisation and increased embodiment compared to other-touch.

Next, we more comprehensively assessed how the sense of body dis-ownership was altered by varying the delay at multiple steps, from 0-594ms, allowing for the calculation of individual thresholds of sensitivity to multi-sensory disruption (see Fig. 1B). Mimicking the results from the ‘long’ task, the sense of body dis-ownership increased with larger delays (Fig. 1B). A linear mixed model was specified with embodiment ratings as the outcome variable, and Delay (0-594ms) and Source (Self-touch, Other-touch), and the interaction between the two included as fixed effects. Each of these were included as a random slope in the model along with a by-subject random intercept. Delay was a significant individual predictor, $\:\widehat{\beta\:}=-0.32$, 95% CI $\:\left[-0.40,-0.25\right]$, $\:t\left(88.99\right)=-8.26$, $\:p<0.001$, such that increased levels of delay were associated with reduced feelings of embodiment. The source of the brushstroke was also a significant individual predictor, $\:\widehat{\beta\:}=0.04$, 95% CI $\:\left[0.00,0.08\right]$, $\:t\left(89.03\right)=2.18$, $\:p=0.032$, such that stroking from another person was associated with reduced feelings of embodiment compared to stroking oneself. The interaction term between delay and source was also significant, $\:\widehat{\beta\:}=-0.07$, 95% CI $\:\left[-0.13,-0.01\right]$, $\:t\left(89.05\right)=-2.33$, $\:p=0.022$, driven by greater discrepancies between self- and other-touch at lower levels of delay. Individual variation in embodiment ratings can be observed in the Supplementary Materials (Figure S1). Data from both ‘long’ and ‘threshold’ versions of the mixed-reality task suggest that delays to visual perception of a brushstroke to the arm induce feelings of reduced ownership over the body.

### Sensitivity to multi-sensory disruption

**Fig. 3:**
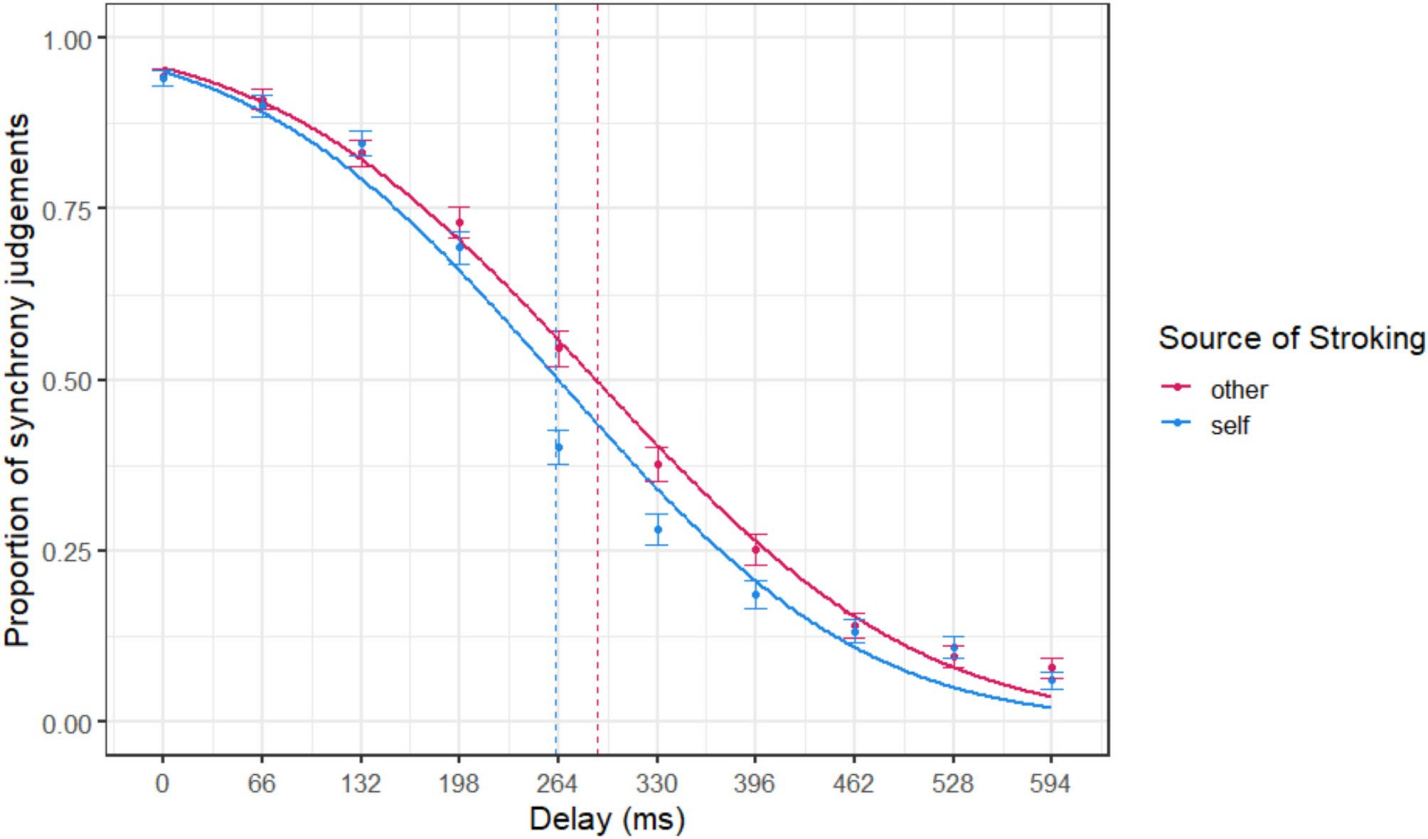
Sensitivity to Multi-Sensory Delays. Fitted Gaussian cumulative psychometric functions for synchrony judgements in response to self- and other-stroking conditions at varying time delays between viewed touch and touch experienced in real time. Vertical dashed lines represent average estimated Point of Subjective Equality (PSE). Points are average proportions of synchrony judgements at each delay level, with error bars representing standard errors from the mean. *N* = 88.

When participants were asked to judge whether the seen and felt sensations were synchronised on the ‘threshold’ task, they tended to begin consistently noticing the delayed visual perception when it was delayed by between 198ms and 330ms (plus the intrinsic delay of the system), as highlighted in Fig. 3. To estimate how sensitive people were to detecting the multi-sensory disruption, the point of subjective equality (PSE) was calculated for each participant by fitting a Gaussian cumulative psychometric function to the proportion of synchrony judgements, separately for self-stroking and other-stroking conditions. Individual PSEs reflect an estimate of the delay that an individual was equally likely to judge as synchronous or asynchronous. Average PSE for other-stroking (M = 290.11ms, SD = 125.94) and self-stroking (M = 261.65ms, SD = 108.21) were significantly different according to a two-sided paired t-test$\:,\:N=88,\:{M}_{D}=-28.46$, 95% CI $\:\left[-52.63,-4.29\right]$, $\:t\left(87\right)=-2.34$, $\:p=0.022$, indicating that participants were more sensitive to the time delay when stroking their own arm, compared to when the stroking was externally-generated.

To investigate if sensitivity to multi-sensory disruption influenced ratings of dis-ownership, PSE for self-stroking and PSE for other-stroking were added as mean-centered fixed effects to the linear mixed model predicting dis-ownership scores on the ‘long’ version of the mixed-reality task. PSE for self-stroking was a significant individual predictor for ratings of dis-ownership, $\:\widehat{\beta\:}=-0.61$, 95% CI $\:\left[-1.00,-0.22\right]$, $\:t\left(80.00\right)=-3.06$, $\:p=0.003$, but PSE for other-generated stroking was not, $\:\widehat{\beta\:}=-0.08,\:95\text{\%}\:\text{C}\text{I}\:\left[-0.42,0.25\right],\:t\left(80.00\right)=-0.49,\:p=0.625,$ suggesting that those with enhanced sensitivity to delay for self-generated stroking also reported greater overall feelings of body dis-ownership on the mixed-reality task.

### Trait dissociation and experimentally induced body dis-ownership

Next, we investigated if experimentally induced experiences of dis-ownership in the mixed-reality paradigm were positively associated with trait measures of dissociation and anomalous experiences. All participants completed the Cambridge Depersonalisation Scale (CDS^42^), a validated measure of the frequency and duration of dissociative experiences, and the Cardiff Anomalous Perceptions Scale (CAPS^39^) a 32-item validated measure of trait anomalous perceptual experiences. The CDS has a total score, and four factors, each capturing a different facet of dissociative experiences: Anomalous Body Experiences, Alienation from Surroundings, Emotional Numbing and Anomalous Subjective Recall^43^. Total score of the CAPS reflects how many items were endorsed by the participant and has subscales assessing the sum of self-reported distress, intrusiveness and frequency of endorsed experiences on 5-point Likert scales. Scores on the CDS and the CAPS scales are presented in Table 2; all were non-normally distributed (see Supplementary Materials). Simple linear regressions (*N* = 87) revealed that average ratings of dis-ownership on the ‘long’ version of the mixed-reality task were significantly associated with total scores on the CDS, $\:{R}^{2}=0.12$, 90% CI $\:[0.03$, $\:0.24]$, $\:F(1,85)=11.82$, $\:p=0.001$, and the CAPS, $\:{R}^{2}=0.08$, 90% CI $\:[0.01$, $\:0.20]$, $\:F(1,85)=7.76$, $\:p=0.007$. Additional regressions found that CDS scores were significantly associated with derealisation, $\:{R}^{2}=0.09$, 90% CI $\:[0.02$, $\:0.20]$, $\:F(1,85)=8.11$, $\:p=0.006$ and deafference, $\:{R}^{2}=0.07$, 90% CI $\:[0.01$, $\:0.18]$, $\:F(1,85)=6.35$, $\:p=0.014$, and CAPS scores were significantly associated with derealisation, $\:{R}^{2}=0.08$, 90% CI $\:[0.01$, $\:0.19]$, $\:F(1,85)=7.20$, $\:p=0.009$, and deafference, $\:{R}^{2}=0.07$, 90% CI $\:[0.01$, $\:0.18]$, $\:F(1,85)=6.65$, $\:p=0.012$. These findings suggest that sensations of dis-ownership, derealisation and deafference reported across conditions were positively related to prevalence of dissociation and anomalous perceptions during daily life.

Spearman correlations explored associations between sensitivity to multi-sensory disruption (PSE) and scores on the CDS and CAPS and their subscales (Table 2). A significant negative correlation was observed between self-touch PSE and CDS, but not between self-touch PSE and CAPS; however, there were significant correlations between self-touch PSE and the distress, intrusiveness and frequency subscales of the CAPS. Examining the correlations between the CDS subscales and self-touch PSE revealed significant negative correlations with the Anomalous Body Experiences and Alienation from Surroundings subscales. In contrast, other-touch PSE only significantly correlated with the Alienation from Surroundings subscale of the CDS. Heightened trait experiences of dissociation and anomalous experiences were therefore associated with an enhanced sensitivity to multi-sensory disruptions of self-generated touch, but not other-generated touch.

**Table 2:** Correlations between trait questionnaires and sensitivity to Multi-sensory disruption.

Variable	Mean (SD)	Median (Range)	Self PSE ($(r_{s} )$	Other PSE ($(r_{s} )$
CDS	36.18 (24.39)	32 (0–118)	−0.26*	−0.19
CDS: ABE	7.74 (6.99)	6 (0–30)	−0.28*	−0.21
CDS: AFS	7.32 (4.87)	6 (0–21)	−0.29*	−0.25*
CDS: EN	7.05 (7.19)	5 (0–28)	−0.17	−0.12
CDS: ASR	9.14 (5.83)	9 (0–30)	−0.16	−0.13
CAPS	6.25 (4.98)	6 (0–28)	−0.20	−0.02
CAPS: distress	14.64 (13.35)	13 (0–82)	−0.24*	−0.03
CAPS: intrusiveness	15.9 (14.19)	13 (0–77)	−0.23*	−0.01
CAPS: frequency	12.44 (12.43)	10 (0–69)	−0.24*	−0.02

Descriptive statistics from full *N* = 100 sample, correlations sample size *N* = 88. Correlation statistics are Spearman correlations. CDS = Cambridge Depersonalisation Scale, ABE = Anomalous Body Experiences, AFS = Alienation from Surroundings, EN = Emotional Numbing, ASR = Anomalous Subjective Recall, CAPS = Cardiff Anomalous Perceptions Scale, SD = Standard Deviation, PSE = Point of Subjective Equality, $\:{r}_{\text{s}}$ = Spearman correlation coefficient.

*p < 0.05.

### Interoception, tactile acuity and phenomenological control

Our final set of pre-registered analyses examined if sensations of body dis-ownership induced by multisensory disruption were negatively associated with both interoceptive and tactile perceptual sensitivity, and positively associated with the ability to adjust experience to match suggestion (phenomenological control). A heartbeat discrimination task^39,40^ assessed interoceptive ability. Participants determined whether sequences of auditory sounds were played in- or out-of-synchrony with their own heartbeats and rated confidence in their responses. Three related, yet distinct, measures of interoceptive ability were calculated^44^. *Interoceptive accuracy* was calculated as the $\:\text{p}\text{e}\text{r}\text{c}\text{e}\text{n}\text{t}\text{a}\text{g}\text{e}\:\text{o}\text{f}\:\text{c}\text{o}\text{r}\text{r}\text{e}\text{c}\text{t}\:\text{r}\text{e}\text{s}\text{p}\text{o}\text{n}\text{s}\text{e}\text{s}$, *interoceptive confidence* was calculated as the average confidence rating on a continuous scale from 0 (Not confident at all) – 1 (Very confident) and *interoceptive awareness* was calculated from the correspondence between accuracy and confidence. The latter metacognitive measure was derived using maximum likelihood estimation to determine *meta-d’* for each individual^45^. Tactile acuity was assessed via performance at judging the orientation (horizontal vs vertical) of specialized grooved objects with decreasing (and therefore less easily detectable) widths between grooves^41^. Phenomenological control was assessed with the SWASH^35^ paradigm in which participants listened to several perceptual suggestions (e.g. imagine heavy bowling ball in your outstretched hands). Their response to each suggestion was judged objectively by the participant, who was asked to judge their own response from a bystander’s perspective (e.g. did they think that their hand drop by 6 inches? ), and subjectively through self-reported vividness of the suggested perception.

To explore unitary associations between interoception, tactile acuity and phenomenological control, we first examined correlations between these variables and the self-reported dissociation ratings from the ‘long’ version of the mixed-reality (Table 3). Interoceptive accuracy was found to be significantly associated with greater ratings of derealization and de-afference, whereas neither interoceptive confidence nor interoceptive awareness were significantly correlated with self-reported dissociation ratings. Heightened pleasantness ratings were significantly positively associated with phenomenological control, but negatively associated with tactile acuity. Then, we sought to determine if either interoception, tactile acuity or phenomenological control predicted the extent to which multi-sensory disruption led to a sense of body dis-ownership. These scores were entered as mean-centered fixed effects to the model predicting dis-ownership scores on the ‘long’ version of the mixed-reality task, along with the additional two-way interactions with the Delay and Source conditions. None of the additional effects were individually significant.

**Table 3:** Associations between interoceptive variables and dissociation scores.

Variable	*N*	Mean (SD)	Disownership	Derealisation	De-afference	Embodiment	Pleasantness
Interoceptive Accuracy	85	59.9 (12)	0.12	0.26*	0.22*	0.05	−0.06
Interoceptive Confidence	85	0.54 (0.17)	0.01	0.05	0.13	0.11	0.16
Interoceptive Awareness	80	0.45 (0.78)	0.10	0.19	−0.02	0.03	−0.13
Tactile Accuracy	86	3.08 (1.56)	−0.07	−0.03	0.14	−0.05	−0.26*
SWASH Score	81	1.46 (0.56)	0.03	−0.02	0.17	−0.03	0.36***

SD = Standard Deviation. Statistic is Pearson’s correlation coefficient. Interoceptive accuracy is percent correct responses on heartbeat discrimination task, interoceptive confidence is average confidence rating and interoceptive awareness is meta-d’, the correspondence between accuracy and confidence estimated with maximum likelihood estimation. N refers to the total number of participants who had completed both the ‘long’ version of the mixed-reality task and each of the 5 variables. SWASH = Sussex-Waterloo Scale of Hypnotisability.

***p < 0.001, *p < 0.01 *p < 0.05.

### Cardiac signatures during multi-sensory disruption

**Fig. 4:**
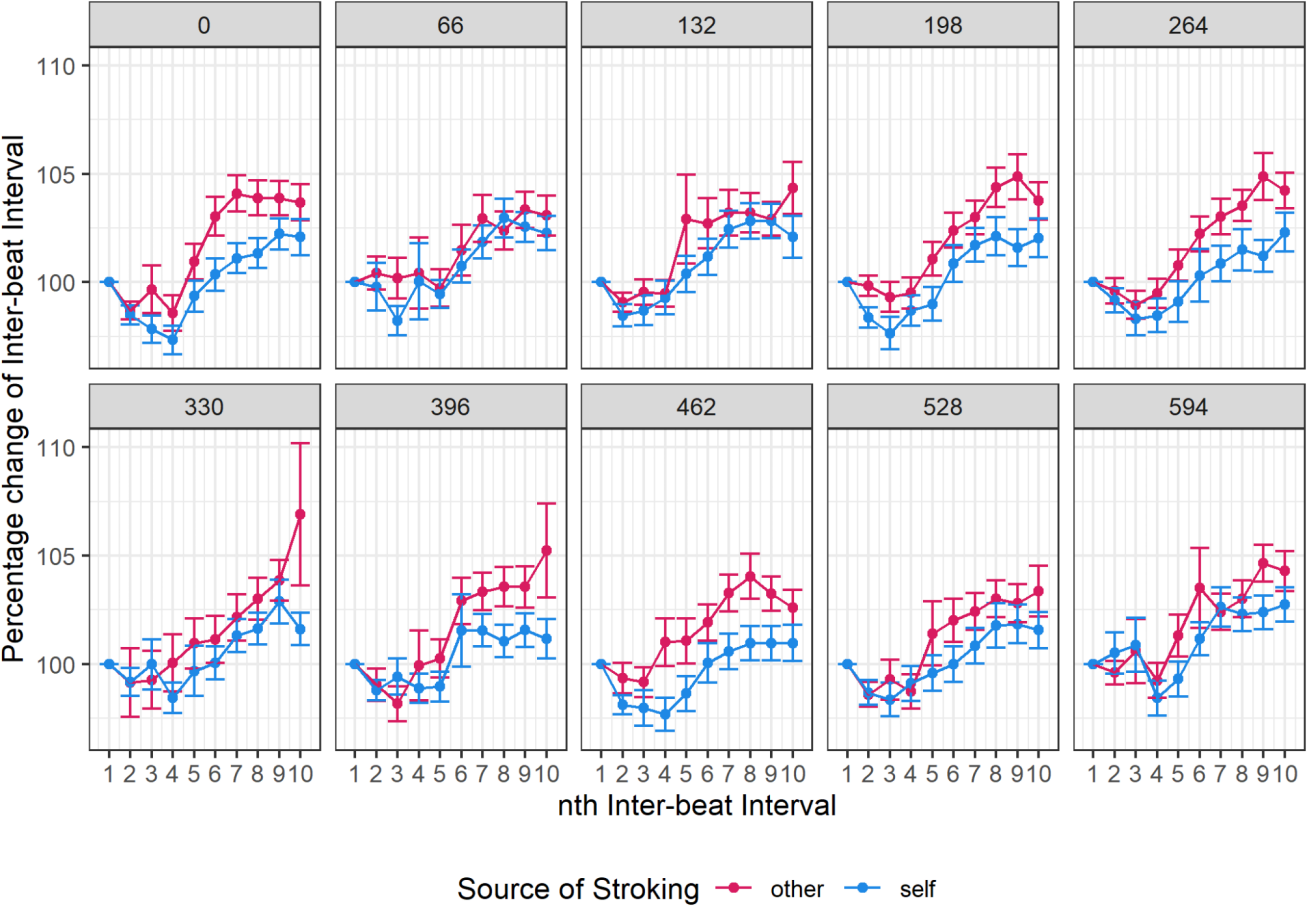
Cardiac response to Mixed-Reality task. Points display mean percentage change in Inter-beat Interval (IBI) from the 1st IBI recorded during each trial, across the first 10 IBIs recorded in a trial. Each graph displays cardiac data averaged across all trials within each level of delay (0ms to 594ms), and in response to self- and other-stroking. Values of 100% represent no change, while lower values represent heart rate acceleration, and higher values represent heart rate deceleration. Error bars are standard errors from the mean.

For a subsample of participants (*N* = 58), heartbeats were recorded using electrocardiography (ECG) during the mixed-reality task, therefore we conducted an exploratory analysis to determine if cardiac signatures varied across conditions and if these variations were related to trait dissociation. After pre-processing, time between heartbeats (inter-beat-intervals, IBIs) during each trial was calculated and expressed as a percentage change from the first IBI on that trial. Increases in this metric reflect deceleration of the heart across the trial, whereas decreases reflect acceleration (Fig. 4).

A linear mixed model (*N* = 53) was conducted to determine if there were any significant differences between conditions in acceleration/deceleration of the heart. Intercepts varied significantly across participants, indicating a linear mixed model was appropriate, SD = 0.0267 CI 95%: [0.022, 0.032], x^2^ change = 1253.26, *p* < 0.001. Predictor variables were IBI (2–10), Delay (0–594ms) and Source (self, other). The full model is reported in Table 3. Both IBI and Source significantly predicted heart acceleration, but level of delay did not (Table 4). From inspection of the graph, it appears that heartbeats initially accelerated, but then decelerated as the trial progressed, with a greater deceleration of heart rate in the “other” condition.

**Table 4:** Linear Mixed-Model exploring impact of delay, source and IBI on cardiac acceleration.

Variable	Value	Std.Error	DF	t-value	*p*-value
(Intercept)	0.98	0	34,622	230.92	0.00
IBI	0.01	0	34,622	23.71	0.00
Delay	0.00	0	34,622	−0.23	0.82
Source	−0.01	0	34,622	−9.05	0.00

IBI = Inter-beat Interval. IBI ranged from 2–10, Delay ranged from 0-594ms in steps of 66ms. Source was self vsersus other-stroking.

**Fig. 5:**
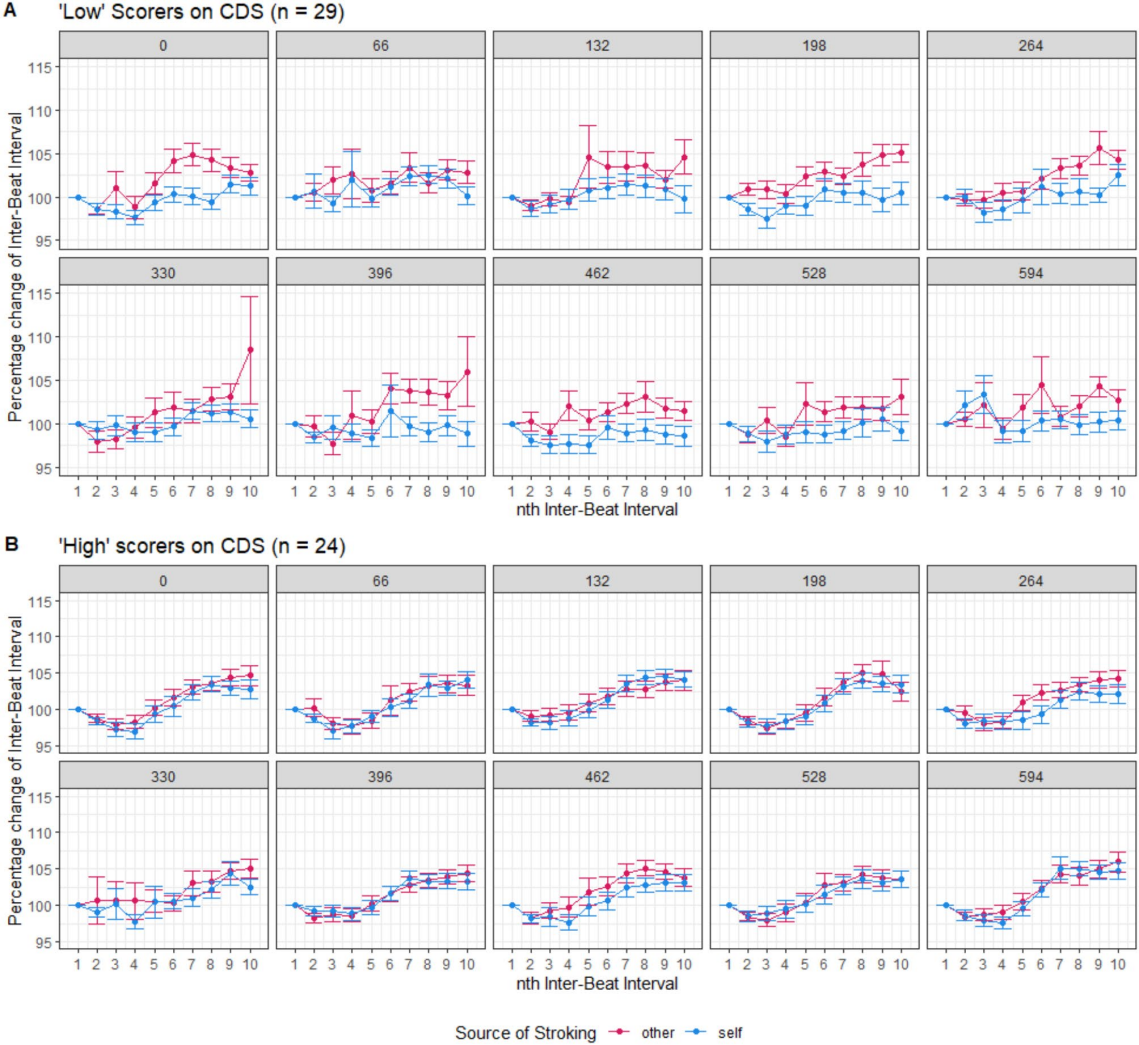
Cardiac response in those with high and low trait dissociation. Graph A represents cardiac response from ‘low’ scorers on the Cambridge Depersonalisation Scale (CDS), on the basis of a median split, graph B represents cardiac response from ‘high’ scorers. Points display mean percentage change in Inter-beat Interval (IBI) from the 1st IBI recorded during each trial, across the first 10 IBIs recorded in a trial. Each graph displays cardiac data averaged across all trials within each level of delay (0ms to 594ms), and in response to self- and other-stroking. Values of 100% represent no change, while lower values represent heart rate acceleration, and higher values represent heart rate deceleration. Error bars are standard errors from the mean.

To explore how heart response was associated with dissociation, we conducted a median split on participants based on total score on the Cambridge Depersonalisation Scale. Median CDS was 32, with 29 participants scoring under this value (“Low” CDS group, Mean = 17.07, SD = 10.39) and 24 participants scoring above this value (“High” CDS group, Mean = 58.50, SD = 23.34). The “High” CDS group should be considered as a group who were more prone to dissociative experiences, rather than a subgroup with dissociation. Figure 5 shows the same average change in heart acceleration low and high CDS groups. There appeared to be less of an effect of the source of stroking for the high CDS group, and when CDS grouping was included as an additional predictor in the LMM in Table 4, it significantly interacted with source, $\:b$ = -0.0128, $\:t$(34621) = -4.62, $\:p$ < 0.001. Thus, cardiac responses, which were normally distinct for self- and other-touch, showed no such distinction in participants who were more prone to dissociation.

## Discussion

The present study employed a mixed-reality approach to introduce a variable time delay to visual perceptions of touch in a non-clinical sample. In line with previous research^20,21^, we found that feelings of dis-ownership from the body increased monotonically with lengthening delay between seen and felt sensations of a brushstroke to the arm (Hypothesis 1a), and extended previous research with the novel finding that greater self-reported dissociation and anomalous experiences in daily life were associated with greater elicited sensations of dis-ownership (Hypothesis 2). Though there was no significant difference in feelings of dis-ownership between self-touch and other-touch (Hypothesis 1b), we found that trait dissociation was significantly associated with greater sensitivity in perceiving the delay between seen and felt sensations of self-generated touch. Interoceptive accuracy, or the capacity to accurately detect heartbeats at rest, was not significantly associated with body dis-ownership (Hypothesis 3), but was associated with greater task-induced feelings of de-afference (feelings of numbness) and derealisation (separation from reality). Neither tactile acuity nor ability to adapt perception according to suggestion (phenomenological control) were significantly related to feelings of dis-ownership (Hypotheses 4 & 5). Finally, exploratory analysis of heart data in a subsample of participants revealed altered acceleration/deceleration of the heart during other-touch compared to self-touch, but this distinction was less apparent in people who were more prone to dissociation.

Individuals who reported more dissociative experiences during their daily life also experienced stronger sensations of experimentally induced body dis-ownership. These findings add to previous research showing that trait levels of unusual experiences are associated with susceptibility to the rubber hand illusion^46^ and to alterations of other characteristics of the bodily self, such as peri-personal space^47^. Evidence that disrupted integration of visuo-tactile experiences can elicit sensations akin to dissociation implies that altered multi-sensory integration may be involved in day-to-day dissociative experiences. Successful integration of incoming sensory information about the body is a key building block for the sense of one’s own body^10^, based largely on research showing that body ownership can be extended to incorporate a fake hand given appropriately synchronized visuo-tactile sensations^16^. The current findings highlight a direct link between disrupted multi-sensory cohesion and predisposition to dissociation, a transdiagnostic experience^4^ involving feelings of detachment from the body.

Analysis of responses to multiple visuo-tactile delays highlighted that most participants experienced a gradual reduction in the sense of ownership over their own body with increasing delays, rather than a sudden loss of ownership after a certain amount of delay. This finding aligns with causal inference models of the sense of the body, which predict that illusions of body ownership gradually diminish with greater temporal or spatial discrepancies between senses^12,14^. Additionally, people were generally more sensitive to multi-sensory disruptions when stroking their own arm, confirming previous findings^21^. Delayed visual perception of self-touch, compared to other-touch, carries an extra multi-sensory conflict between the sight and sensation of one’s own movement, potentially causing it to be more noticeable.

Interestingly, individuals with higher trait dissociation noticed the asynchrony between vision and self-initiated touch at smaller delays than people with lower trait dissociation. This suggests that individuals more prone to dissociation may be less likely to integrate senses to create an integrated percept, contributing to a fragmenting of senses that might result in dissociative experiences. Different senses, each arriving to and being processed by the brain at different timescales, must be integrated together in order to form a coherent percept of the body^10^ and the body in the world. Our findings suggest that dissociation may be linked to difficulties in successfully integrating senses across modalities. Previous work has also demonstrated altered multi-sensory integration in those pre-disposed to unusual bodily experiences. A non-clinical group more prone to out-of-body experiences (OBEs), showed evidence of embodiment over a rubber hand even when brushing to the real and rubber hand were asynchronous in time^48^. OBEs may stem from reduced sensitivity to time discrepancies between seen and felt touch, resulting in even temporal disparate touch being perceived as synchronous, thus causing embodiment over the rubber hand. Sensitivity to multi-sensory disruption was not assessed in this previous study. Nevertheless, there may be important nuances in how multi-sensory integration is altered in different unusual bodily experiences that should be explored further.

Greater sensitivity to disruption of self-action may alternatively arise from greater awareness of the sensations accompanying self-generated sensations. The sensory consequences of self-action are typically attenuated, as these sensations add little information to internal simulations or predictions of the motor act^49^. It is this process that prevents tickle sensations from accompanying self-touch: the tactile experience is fully predictable, and so the tickle sensation is attenuated^50^. Models of psychosis have often emphasized impaired internal simulations of self-action^51,52^, which has since been expressed as reduced precision of prior beliefs in predictive coding models of psychosis^53,54^. Both approaches make the prediction that sensory consequences of self-actions may not be attenuated in people with psychosis, which is supported by evidence that people with schizophrenia are more successful in tickling themselves^55^. A similar over-awareness of self-generated actions may be involved in dissociative sensations^56^, given that self-disturbances are argued by some to be the core feature of psychosis symptoms^57^. Our findings of heightened sensitivity to disruption of self-touch in people more prone to dissociation further supports this theory.

Greater sensitivity to self-touch may also arise from greater sensitivity to visuo-motor conflicts, given that self-touch involved movement of the arm to deliver a brushstroke. Past research implies a minimal role for visuo-motor conflicts in noticing a temporal asynchrony during self-touch in the mixed-reality task. Similar sensitivity to disruption was observed between a typical self-touch condition and a self-touch condition where vision of the moving hand was occluded, thus removing the visuo-motor conflict^21^. Future research may explore how dissociation relates to these different self-touch conditions to clarify the relative contribution of these different mechanisms to dissociation.

The delay between seen and felt sensations of touch also resulted in feelings of numbness (de-afference) and feelings of separation from reality (derealisation), which were both significantly positively associated with interoceptive accuracy. We expected interoceptive accuracy to be negatively associated with induced experiences of dissociation because greater interoceptive accuracy has previously been shown to be protective against the altered sense of the body induced by the RHI^26^, and tends to be reduced in people who regularly experience dissociation^32,33^. However, the evidence base for relationship between the RHI and interoceptive accuracy is mixed, with more recent studies finding no significant association between the two^23,58,59^. Moreover, studies finding reduced interoception in patient samples with high dissociation have not controlled for medication^32,33^,. which impacts both physiological activity and interoceptive sensation and may have confounded results^60^. Feelings of numbness over the body are commonly reported in body ownership illusions^61^, and people with higher interoceptive accuracy tend to report greater spontaneous sensations of numbness (i.e. not triggered by an external touch) than people with low interoceptive accuracy^62^.Although historically interoception referred solely to the sensing of visceral sensations (e.g. cardiac, respiratory, gastric), more recent definitions encompass the sensing of touch which convey information about the physiological state of the body^27^, such as affective touch, tickle and itch, following evidence that tactile receptors for these types of touch project to interoceptive brain regions^63^. Feelings of numbness in response to the visuo-tactile delay in our task may therefore be partly interoceptive sensations, which would explain their association with interoceptive accuracy.

No significant association was observed between experimentally induced dis-ownership and phenomenological control, suggesting that the ability to adapt experience according to ‘top-down’ suggestions had minimal influence on the induced sensations. This contrasts with previous research which found a positive association between phenomenological control and embodiment in the rubber hand illusion^32^, though see^33,34^. Our lack of association may be due to the use of a shortened version of the measure of phenomenological control, which used only 4 items from the original 10-item measure^35^. Nevertheless, it may be that feelings of detachment from one’s own body in the mixed reality task are less influenced by suggestibility than the extension of body ownership that occurs in the rubber hand illusion. The fake hand in the RHI offers a salient cue to participants that they may experience an altered sense of their own body. In contrast, during the mixed-reality task participants are observing their own body, so may hold fewer expectations about how the manipulation of visuo-tactile synchrony should influence their sense of body ownership. Suggestibility may therefore play a reduced role in the intensity of the experience compared to the RHI. Indeed, if dissociation involves a heightened awareness of self^56^, then we might predict the opposite relationship; that greater dissociation would be related to a reduced ability to adapt ongoing experience to ‘top-down’ suggestions, but further research using the full version of the SWASH may be required to clarify these relationships.

An exploratory analysis of heart data recorded during the mixed-reality task revealed distinct cardiac signatures between self- and other-touch reflecting altered profiles of heart acceleration/deceleration across time. A median split of participants into “high” and “low” dissociation groups suggested that this distinction was less apparent for the “high” group. This finding implies that people who are highly prone to dissociation may demonstrate similar physiological responses to self- and other-generated sensations. This finding resonates with the explanation that dissociation is related to heightened awareness of the sensory consequences of self-action^49,52,56^. Given the exploratory nature of the cardiac analysis, and the limitations inherent in interpreting results from median split samples^64^, this data should be interpreted with caution. However, it does highlight a potential area of interest for future research.

In conclusion, the present study demonstrated that delaying visual perception of tactile sensations with mixed-reality can result in a loss of ownership over the body. For the first time, we demonstrated that the strength of these induced sensations were positively associated with trait dissociative experiences in a non-clinical population. This implies that induced and trait dissociation may both be caused by a separation of normally integrated sensations. Furthermore, those with heightened trait and induced dissociative experiences were better able to notice the delay, but only for self-generated tactile sensations. This finding further points towards a ‘fractioning’ of the senses in dissociation, with dissociative experiences potentially linked to a disrupted ability to bind senses together to form a coherent percept of the body. That this was unique to self-generated sensations also aligns with theories suggesting that dissociative experiences stem from over-attending to self-generated sensations. Taken together, these findings implicate two mechanisms which may be altered in dissociation: disruptions to multi-sensory integration processes, and a heightened awareness of the sensory consequences of self-generated sensations.

## Methods

Ethical approval for the research study was obtained from the University Research Ethics Committee at the University of Sussex. All methods were performed in accordance with the relevant guidance and regulations for research involving human participants, including those set out by the University Research Ethics Committee and the Declaration of Helsinki.

### Participants

An a priori power analysis was used to calculate target sample size. In previous research, associations between subjective feelings of embodiment on the RHI and psychosis-proneness ranged from *r* = 0.35 to *r* = 0.42^65^, and associations with positive schizotypy were reported as *r* = 0.28^46^. G*Power software^66^ was therefore used to calculate suggested sample size, given an alpha level of 0.05 and a cautious estimate of correlation of *r* = 0.28, which suggested a sample size of *N* = 75 to achieve 80% power. On this basis, 100 participants were recruited to take part to allow for potential drop-out. All participants were required to be aged 18–65 and have no historic or current diagnosis of a psychiatric, developmental or neurological disorder. Participants were recruited via convenience sampling with posters displayed around a UK university campus and advertisements on a University experiment management system. Written informed consent was gathered from participants prior to the beginning of the study.

### Measures

#### Mixed-reality task

The mixed-reality task was conducted in two parts, the “threshold” part and a subsequent “long” part. In both parts, participants wore a Head-Mounted Display (HMD; Oculus Rift). A webcam with a wide field-of-view was mounted on the HMD, and the webcam video feed was displayed on the HMD during the task to provide participants with an online first-person perspective of their body and the surrounding environment. Participants were seated with their left arm resting on a table. For a subsample of participants (*n* = 58), heart rate was recorded using electrocardiography (ECG) which became available part way through the study, with three wired sensors attached to the chest and lower back.

The threshold part of the mixed-reality task consisted of two sets of forty trials in which the participant’s arm and hand were stroked with a brush for seven seconds. This brushstroke was delivered by the researcher for one set of forty trials (Other condition), and by the participant themselves for the other set of forty trials (Self condition), the order of which was counterbalanced between participants. 2–3 brushstrokes were conducted per trial, moving from the middle of the forearm to the fingertips. The experimenter demonstrated how to conduct the stroking in the self-stroking condition and observed the participant when carrying out the touch and corrected them if they deviated from the demonstrated touch, to ensure that stroking characteristics were the same in other- and self- conditions. Across the study, two different computers were used, each of which had a slight difference (14ms) in intrinsic delay of the system, with an average intrinsic delay of 134.23ms. There was no significant difference in participant’s sensitivity to the delay between the two computers, suggesting that this did not impact performance on the task (see Supplementary materials). In addition to the intrinsic delay of the system, a time delay ranging from 0-594ms in steps of 66ms was introduced to the video feed on the HMD, with each delay time repeated four times in a random order. After each trial, participants reported whether the sensations they saw and felt were synchronous, and rated their sense of ownership over their arm and the pleasantness of the experience on two continuous visual analogue scales ranging from 0 to 1, with 0 being “strongly disagree” and 1 being “strongly agree.”

The long part of the mixed-reality task consisted of four trials in which the participant either stroked their own arm (Self condition) or the researcher stroked the participants arm (Other condition) for 60 s. The stroking was delivered from the forearm to the fingertips, at the same pace used for the threshold task. The visual feed on the HMD was either not delayed (Synchronous condition) or was delayed by 1 s (Asynchronous condition), after accounting for intrinsic delay. This resulted in four trial types: self-delay, self-no delay, other-delay, other-no delay, the order of which was counterbalanced across participants. After each trial, participants rated their experience on nine visual analogue scales (see Table 5) ranging from 0 to 1, with 0 being “strongly disagree” and 1 being “strongly agree.”

**Table 5:** Questionnaire items used in the ‘long’ version of the mixed-reality task.

Item	Sometimes I felt…	Factor
1.	Alienation from my body	Disownership
2.	As if my body did not belong to me anymore	Disownership
3.	As if my body was numb	Deafference
4.	As though the experience of my hand was less vivid than normal	Deafference
5.	As if the body I saw was my own	Embodiment
6.	As if I could move the seen body	Embodiment
7.	As if I were not real or as if I were cut-off from the world	Derealisation
8.	As if my surroundings felt detached or unreal	Derealisation
9.	That the experience was pleasant	Pleasantness

Items 1–6 adapted from previous research using the Mixed-Reality task which used a Principal Component Analysis to identify Disownership, Deafference and Embodiment as distinct yet related experiences reported in response to the task^20^. Items 7–8 adapted from Cambridge Depersonalisation Scale ^42^.

#### Interoception

Interoception was assessed with the heartbeat discrimination task^39,40^. Heartbeats were recorded using a pulse oximeter attached to the left index finger (‘soft’ mount PureLight sensor; Nonin Medical Inc, MN, USA). On each of sixty trials, ten computer-generated auditory tones were presented either in synchrony with the participant’s heartbeat (during the cardiac ejection period, to coincide with the rise of the pulse pressure wave and overlapping with t-wave, synchronous condition) or out of synchrony with their heartbeat (asynchronous condition, between heartbeats). After each trial, the participant indicated whether they thought the tones were in time or out of time with their heartbeat. The participant rated their confidence in their response on a continuous visual analogue scale ranging from 0 (“Not confident at all”) to 1 (“Very confident”). Three outcome measures were calculated, representing interoceptive accuracy (percentage of correct responses on the task), confidence (average confidence rating) and metacognitive awareness, the correspondence between accuracy and confidence that is also termed interoceptive insight^27^, and calculated using maximum likelihood estimation to determine meta-d’ for each individual^45^ (code: http://www.columbia.edu/~bsm2105/type2sdt/).

#### Tactile acuity

 The tactile acuity of individuals was assessed using a tactile discrimination task^41^. This task used eight grooved, dome shaped plastic objects (JVP tactile domes, Stoelting Co., Wood Dale, IL, USA) with varying square-wave grating widths (3, 2, 1.5, 1.2, 1, 0.75, 0.5 and 0.35 mm). Each object was applied twelve times to the index finger of the left hand. The object was positioned so that the grating widths were parallel to the index finger on half of the trials and perpendicular on the other half. Without looking, participants stated the orientation of the gratings (horizontal or vertical) and rated their confidence in their answer on a visual analogue scale ranging from 0 (“Not confident at all”) to 1 (“Very confident”). The task was stopped if a participant correctly answered fewer than 70% of the trials for a given object. The outcome measure of tactile acuity was calculated as the number of objects for which accuracy was greater than 70% (0–8).

#### Phenomenological control

 A short version of the Sussex-Waterloo Hypnotisability Scale (SWASH; https://osf.io/wujk8/) assessed phenomenological control. During the task, the participant listened to an audio recording which encouraged them to imagine four situations: (1) holding a heavy bowling ball in their outstretched hand, (2) the presence of a buzzing mosquito, (3) that their resting hand is very heavy and (4) that a picture of 3 balls displayed on the computer screen only has 2 balls. Afterwards, participants rated how vividly they experienced each situation on Likert scales from 0 to 5, with higher scores indicating a more vivid response. The mean rating of all the situations was taken as the subjective score. Participants also reported whether they objectively responded to the suggestions (from a bystander’s perspective) according to pass or fail criteria. For example, if the participant reported lowering their hand by at least six inches in response to imagining a bowling ball in their hand, they are classified as having objectively responded to that suggestion. The number of passes was taken as the objective score (from 0 to 4). The outcome measure is a combined score, calculated as the mean value of the subjective and objective scores (with the subjective score multiplied by 0.8 to be on a 0–4 scale).

#### Questionnaires

Each questionnaire was programmed in JavaScript using the jsPsych package^67^. Age, gender, ethnicity, years of education, frequency of alcohol and cannabis use, and handedness^68^ were recorded in a demographics form.

#### Cambridge Depersonalisation Scale (CDS; Sierra & Berrios, 2000)

The CDS is a 29-item questionnaire, designed to assess experiences of depersonalisation. Each item describes an unusual experience (e.g. “Parts of my body feel as if they didn’t belong to me”). The participant is asked to indicate the frequency at which the experience occurred over the past 6 months and the duration of the experience. Frequency is rated on a 5-point scale from 0 (Never) to 4 (All the time) and duration is rated on a 6-point scale from 1 (A few seconds) to 6 (More than a week). The outcome measure was the total summed score of the items (0-290). The CDS has a total score, and four factors, each capturing a different facet of dissociative experiences: Anomalous Body Experiences, Alienation from Surroundings, Emotional Numbing and Anomalous Subjective Recall^43^.

#### Cardiff Anomalous Perceptions Scale (CAPS; Bell, 2006)

The CAPS is a 32-item questionnaire designed to assess unusual perceptual experiences. Each item describes an unusual sensation or perception (e.g. “Do you ever notice that sounds are much louder than they normally would be?”), and the participant indicates whether they have ever had that experience. If the participant indicates that they have had the experience, they rate the experience on three Likert scales (ranging from 1 to 5), which assess the frequency, distress and intrusiveness of the experience. The primary outcome measure was the total number of endorsed items.

### Procedure

After giving consent to take part and completing a demographics form, participants first completed a somatic signal detection task, the results of which are reported elsewhere (Moffatt et al., in prep). Participants then completed the questionnaires, followed by the interoception task and the mixed-reality task.

### Statistics

The study protocol, key hypotheses, variables of interest and analysis plan were pre-registered at https://osf.io/qh543/ on April 2nd, 2020, after the end of data collection (February 2020) but prior to any analysis of the data. All analyses were carried out in R (R Core Team, 2022) and Rstudio (Rstudio Team, 2022), using the following packages: *here*^69^, *flextable*^70^, *cowplot*^71^, *quickpsy*^72^, *Hmisc*^73^, lme4^74^, lmerTest^75^ and the *tidyverse* set of packages^76^.

The findings from the initially pre-registered regression approach are reported in the supplementary materials for completeness. The analytical approach reported below takes a linear mixed model approach to best account for the repeated measures nature of the task design. The findings from the two approaches are highly similar.

#### Hypothesis 1a

Feelings of dis-ownership will be heightened when visual perception of a brush stroke delivered to the arm is delayed.

#### Hypothesis 1b

Feelings of dis-ownership will be reduced when the participants stroke their own arm, compared to being stroked by another person.

For the “long” part of the mixed-reality task, a linear mixed model was conducted, with ratings of dis-ownership entered as the outcome variable, with Delay (No delay, Delay) and Source (Self, Other), as well as the interaction between the two, added as fixed and random effects, and a by-subject random intercept. For the “threshold” part of the mixed-reality task, a linear mixed model was specified with ratings of embodiment (as an inverse measure of dis-ownership) as the outcome variable, with Delay (0-594ms), Source (Self, Other), and the interaction between the two entered as predictor variables. The model included a random by-subject intercept and random slopes for Delay, Source and the interaction between the two.

Analyses were conducted to determine if individual sensitivity to multi-sensory disruption differed according to the source of stroking, and to examine how sensitivity to delay was associated with induced feelings of body dis-ownership. The Point of Subjective Equality (PSE) represents sensitivity to the disruption, and is an estimate of the amount of delay at which an individual was maximally uncertain about whether the seen and felt sensations were in synchrony. PSEs were calculated by fitting a Gaussian cumulative psychometric function to participants synchrony judgements across each level of delay, separately for self- and other-stroking. Psychometric fits were conducted with the ‘*quickpsy*’ R package using 1000 bootstrapped samples. Average PSE was compared between Self and Other conditions with a paired t-test. PSE for self-stroking and PSE for other-stroking were then entered as mean-centered fixed effects to the linear mixed model predicting dis-ownership ratings on the ‘long’ task, to determine if sensitivity to multi-sensory disruption was associated with induced feelings of dissociation.

#### Hypothesis 2

Sensations of dis-ownership on the mixed-reality task will be positively associated with scores on dissociative experience questionnaires.

To determine the relationship between trait and induced feelings of dissociation, two simple linear regressions were calculated with scores on CDS and CAPS questionnaires entered as the outcome variables, and mean ratings of dis-ownership on the “long” part of the mixed-reality task as the predictor variable for each regression. Additionally, correlations were computed between the trait anomalous experience measures and the PSE for self-stroking and the PSE for other-stroking, to determine if sensitivity to the multi-sensory disruption was associated with trait dissociation.

#### Hypothesis 3

Interoceptive ability will be negatively associated with sensations of body dis-ownership.

To determine the influence of interoception on induced sensations of dis-ownership, the outcome measures of interoceptive accuracy, interoceptive confidence and interoceptive awareness derived from the Heartbeat Discrimination task were entered as mean-centred fixed effects to three separate linear mixed models for the “long” part of the mixed-reality task, along with the additional two- and three-way interactions with Delay and Source.

#### Hypothesis 4

Individual differences in the ability to sense bodily signals that arise externally (tactile acuity) will be negatively associated with reported feelings of body dis-ownership.

To determine the influence of tactile acuity on induced sensations of dis-ownership, the tactile acuity score was entered as a mean-centred fixed effect to the linear mixed model for the “long” part of the mixed-reality task, along with the additional two- and three-way interactions with Delay and Source.

#### Hypothesis 5

Individual differences in the ability to exert top-down control over perception (phenomenological control) will be positively associated with reported feelings of body dis-ownership.

To determine the influence of phenomenological control on induced sensations of dis-ownership, the SWASH score was entered as a mean-centred fixed effect to the linear mixed model for the “long” part of the mixed-reality task, along with the additional two- and three-way interactions with Delay and Source.

### Heart analysis

Exploratory analyses were also conducted on heart rate data recorded during the mixed-reality task. For the “threshold” version of the task, a metric of acceleration/deceleration of the heart rate was calculated.

A peak detection algorithm in MATLAB smoothed the raw ECG data with a Savitsky-Golay filter and identified heartbeat peaks in the recordings, and these were visually inspected to ensure heartbeats were recorded accurately. Time between peaks, or inter-beat intervals (IBIs) were calculated, and the first IBI of each trial was taken as the baseline IBI for that trial. IBIs larger than 3 standard deviations from the average IBI were excluded as outliers. Percentage change from the baseline IBI was then calculated for each subsequent IBI (up to the 10th IBI in a trial, as more than 10 IBIs in a single trial occurred rarely), a metric which reflects acceleration/deceleration of the heart rate across the trial, with increases reflecting deceleration and decreases reflecting acceleration. A linear mixed-model was then conducted, with percentage change from IBI as the dependent variable and Source of stroking (Self, Other), Delay (0-594ms) and IBI (2–10) included as predictors.

To explore how heart responses related to dissociation, a median split based on CDS scores was carried out to separate participants into “high” and “low” dissociation-prone groups. CDS grouping was included as an additional predictor in the linear mixed model.

## Electronic supplementary material

Below is the link to the electronic supplementary material.

Supplementary Material 1

Supplementary Material 2

## Data Availability

The datasets generated during and analysed during the current study are available in the Open Science Framework repository linked to this study: https://osf.io/qh543/ with the identifier doi:10.17605/OSF.IO/QH543.

## Code availability

The custom code used to analyse the data in the current study are available in the Open Science Framework repository linked to this study: https://osf.io/qh543/.
