# Supplementary material for: Trait dissociation is associated with dissociative experiences arising from disrupted multisensory integration
Source: Sci Rep. 2025 Apr 12;15:12553. doi: 10.1038/s41598-025-97320-9 (PMC11993574; doi:10.1038/s41598-025-97320-9)
Supplement: Supplementary file 1 — Supplementary Material 1 [file 41598_2025_97320_MOESM1_ESM.docx]

Dissociative experiences arise from disrupted multisensory integration

Supplementary Materials

Jamie A. Moffatt^1,2, *^, Marte Roel Lesur^3^, Bigna Lenggenhager^3, 4^, Marieke L. Weijs^3, 5^, Valerio Maglianella^6^, Hugo D. Critchley^6, 7^, Sarah N. Garfinkel^8, †^, & Kathryn Greenwood^1, 7, †^

*^1^School of Psychology, University of Sussex, Falmer, United Kingdom;
^2^Department of Psychology, Royal Holloway, University of London, United Kingdom;
^3^Department of Psychology, University of Zurich, Zurich, Switzerland;
^4^Department of Psychology, University of Konstanz, Konstanz, Germany;
^5^Department of Health Sciences and Technology, ETH Zurich, Zurich, Switzerland;
^6^Brighton and Sussex Medical School, Falmer, United Kingdom;
^7^Sussex Partnership NHS Foundation Trust, Brighton, United Kingdom;
^8^Institute of Cognitive Neuroscience, University College London, London, United Kingdom;
^*^Corresponding Author; ^†^Joint Senior Author.*

1. **Intrinsic Delay**

Here, we describe the method we used to calculate intrinsic delay of the system. We used two computers, so here we report the intrinsic delay of each computer, and report a comparison of the point of subjective equality to determine if performance differed noticeably between the two computers.

- 1. **Intrinsic Delay Calculation**

Intrinsic delay of the system was calculated by taking a slow-motion video recording of a flashing light, with the flashing light visible both in real time and on the computer display. The video was then visually inspected to record the time delay between the flashing light occurring in real time and the flashing light occurring on the computer display. This was repeated for multiple flashing lights, and the average delay time was taken as the intrinsic delay of the system for each computer. The intrinsic delay of Computer A was estimated to be 126.28ms (SD = 12.59ms) and 142.18ms (SD = 11.54ms) for Computer B, a mean difference of 15.9ms.

- 1. **Comparison of Sensitivity to Delay between Computers.**

For the study, 41 participants completed the study using Computer A and the remaining 59 completed the study using Computer B. Despite the slight difference in intrinsic delay (Mean D = 14ms), Welch Two Sample t-tests found no difference in point of subjective equality between the two groups for either self-stroking (t(81.27) = -0.868, p = 0.388, [-0.06, 0.024]) or other-stroking (t(73.48), p = 0.587, [-0.06, 0.03]) conditions. Point of Subjective Equality is an estimate of the point at which participants are likely to notice the delay. This suggests that the difference in intrinsic delay time between the two computers used in the study was not large enough to produce statistically meaningful differences in participant’s ability to notice the time delay.

1. **Missing or Excluded Data**

Of the 100 participants, some participants were unable to complete some tasks, or their responses were discarded from the final analyses following inspection of their data. The amount of participants with missing or excluded data and the reason for their exclusions are listed here.

For the ‘long’ version of the mixed-reality task, discomfort with using mixed-reality (N=8) and technical issues (N=5) prevented some participants completing this part of the study. For the ‘threshold’ version of the mixed-reality task, some participants reported discomfort with mixed-reality (N=6) or experienced technical issues on the day of testing (N=4), which prevented completion of this part of the study. All participants completed the ‘threshold’ version first, and the ‘long’ version second, so all participants who felt discomfort with using mixed-reality or experienced technical issues in the ‘threshold’ version also did not complete the ‘long’ version.

Inspection of individual Point of Subjective Equalities (PSEs) calculated from the ‘threshold’ version of the mixed-reality task revealed that a further N=2 participants should be excluded as outliers because their estimated PSE was greater than 2 standard deviations from the group average PSE. On inspection of their data, these two participants judged almost all delay levels as synchronized, an unusual and likely erroneous response pattern which prevented an accurate PSE being calculated for these individuals.

For the interoception task**,** N=3 participants completed less than 50% of trials due to technical issues with the heartbeat recording equipment, and an additional N=1 participant was not able to provide confidence ratings due to a technical error. *Meta-d’* was unable to be calculated for the preceding participants, plus N=1 who provided the same confidence rating on each trial and N=4 who had a $d’$ of 0. A $d’$ of 0 results from an equal hit rate and false alarm rate on the heartbeat discrimination task, meaning that the individual was effectively unable to discriminate between sequences of tones played in synchrony with the heart and tones played out of synchrony with the heart. Meta-d’ cannot be calculated for these cases. Therefore, N = 3 were missing or excluded from analyses involving interoceptive accuracy, N = 4 were missing or excluded from analyses involving interoceptive confidence and N = 9 were missing or excluded from analyses involving interoceptive awareness.

For the tactile acuity task, N=1 participant did not complete the exteroceptive task due to a clerical error on behalf of the researcher. For the phenomenological control task, N = 7 participants were unable to complete the task due to running out of time during the experiment session and N = 1 participants reported being unable to understand the audio recording, therefore the task was stopped.

Of the N=58 participants where heart rate was recorded during the mixed-reality paradigm, a further N=3 participants were removed from the dataset due to noisy recordings resulting in poor differentiation of the R wave, and technical issues for N=2 participants prevented the recording of heartbeat during the threshold task.

1. Individual-Level Data


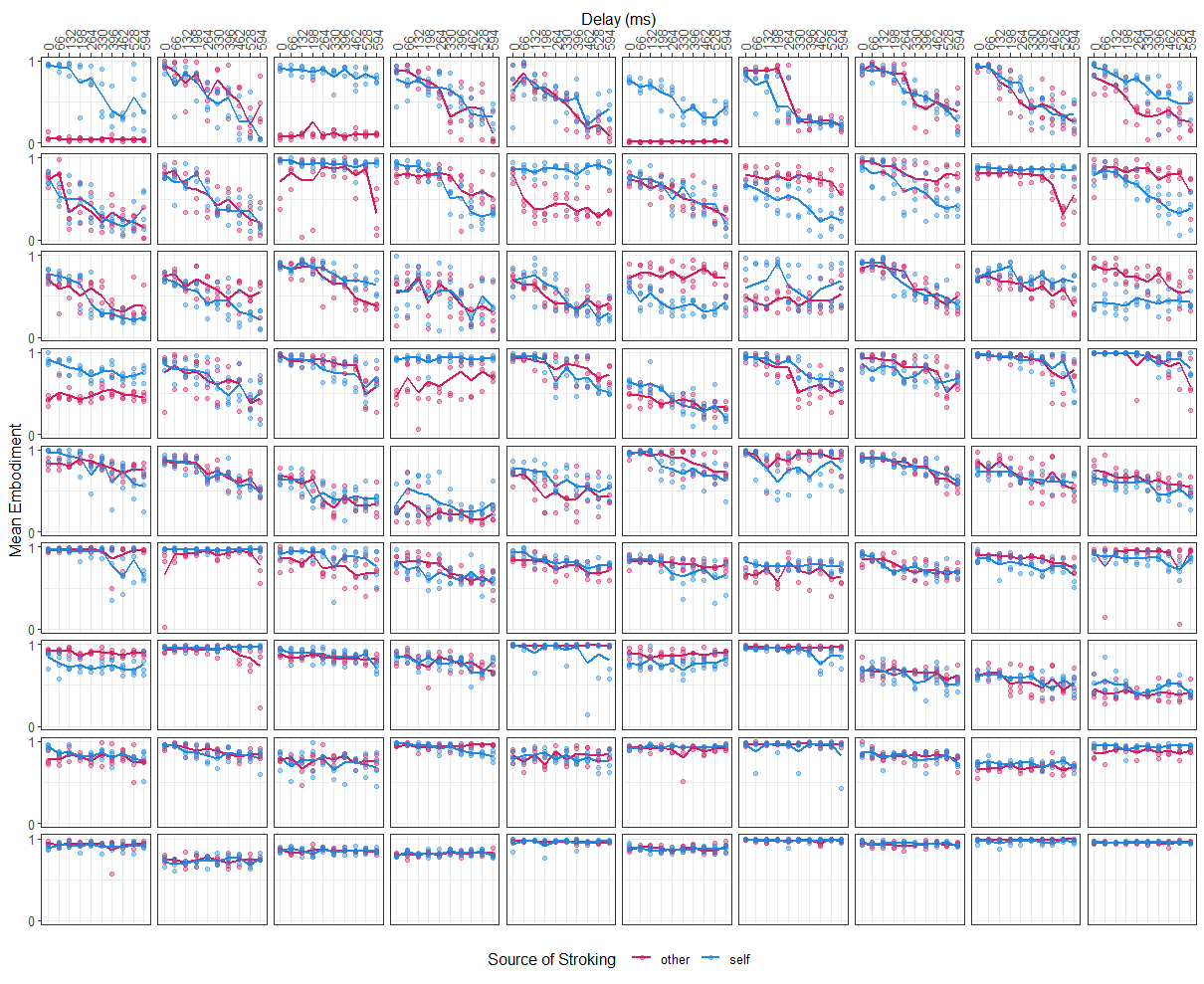


*Figure S1.* **Individual Embodiment Ratings for Threshold Task.** Lines represent mean ratings of embodiment at each delay level ranging from 0-594ms, with points representing individual ratings. Each graph displays data from a single participant (N = 90), with graphs ordered from most to least change in embodiment ratings.

1. **Linear Mixed Model Approach**

As our pre-registered analyses specified using regressions to analyse our hypotheses, they fail to account for the within-participant design of the study.

Here, we have conducted these analyses as Linear Mixed Models, which allows for the specification of a random by-subject intercept. The results are highly similar, but are presented here for completeness.

- 1. **Mixed-Reality Task, “Long” Version**

A multiple regression (N=87) with Delay and Source of stroking as binary predictor variables and average ratings of dis-ownership as the outcome variable was significant, $R^{2}=.32$, 90% CI $[0.25$, $0.39]$, $F(3,344)=55.08$, $p<.001$, but Delay was the only significant individual predictor, $b=-0.32$, 95% CI $[-0.39$, $-0.24]$, $t(344)=-8.69$, $p<.001$. Neither source of stroking, $b=-0.01$, 95% CI $[-0.08$, $0.06]$, $t(344)=-0.28$, $p=.780$, nor the interaction between delay and source, $b=-0.03$, 95% CI $[-0.13$, $0.07]$, $t(344)=-0.52$, $p=.605$, were significant individual predictors. Similar regressions also found that level of delay was also the only significant individual predictor for feelings of derealisation, $b=-0.25$, 95% CI $[-0.32$, $-0.18]$, $t(344)=-6.75$, $p<.001$, de-afference, $b=-0.23$, 95% CI $[-0.29$, $-0.16]$, $t(344)=-6.59$, $p<.001$, embodiment, $b=0.17$, 95% CI $[0.11$, $0.24]$, $t(344)=5.13$, $p<.001$, and pleasantness, $b=0.10$, 95% CI $[0.02$, $0.17]$, $t(344)=2.63$, $p=.009$.

These findings are differ from our findings with a linear mixed model approach reported in the primary manuscript. In the linear mixed model approach, we found additional effects of source for derealization and embodiment ratings as well as a significant interaction between source and delay for pleasantness ratings.

- 1. **Mixed-Reality Task, “Threshold” version**

A multiple regression (N=90) including both Source and Delay as predictor variables significantly explained variance in embodiment ratings, $R^{2}=.10$, 90% CI $[0.08$, $0.12]$, $F(3,1864)=69.90$, $p<.001$, and Delay was a significant individual predictor, $b=-0.32$, 95% CI $[-0.39$, $-0.25]$, $t(1864)=-8.99$, $p<.001$, such that increased levels of delay were associated with reduced feelings of embodiment. The source of the brushstroke was also a significant individual predictor, $b=0.04$, 95% CI $[0.00$, $0.07]$, $t(1864)=2.23$, $p=.026$, indicating that stroking from another person was associated with reduced feelings of embodiment compared to stroking oneself. The interaction term between delay and source was not significant, $b=-0.07$, 95% CI $[-0.17$, $0.03]$, $t(1864)=-1.46$, $p=.145$.

This is similar to our findings from the linear mixed model approach reported within the primary manuscript, with the exception that the linear mixed model approach found a significant interaction between delay and source. On inspection of Figure 2 in the primary manuscript, an interaction seems likely. There is a difference between self- and other-stroking at lower levels of delay, from 0-200ms, with self-stroking resulting in greater reported feelings of embodiment, but this difference was reduced as delay increased.

- 1. **Point Of Subjective Equality**

To investigate if sensitivity to multi-sensory disruption influenced ratings of dis-ownership, PSE for self-stroking and PSE for other-stroking were added as variables to the model predicting dis-ownership scores on the ‘long’ version of the mixed-reality task. PSE for self-stroking was a significant individual predictor for ratings of dis-ownership, $b=0.62$, 95% CI $\left[ 0.35,0.89 \right]$, $t\left( 326 \right)=4.51$, $p<.001$, but PSE for other-generated stroking was not, $b=0.03$, 95% CI $\left[ -0.21,0.26 \right]$, $t\left( 326 \right)=0.22$, $p=.822$, suggesting that those with enhanced sensitivity to delay for self-generated stroking also reported greater overall feelings of body dis-ownership on the mixed-reality task.

This is identical to our findings with a linear mixed model approach reported in the primary manuscript.

- 1. **Associations with mechanisms**

In separate multiple regressions, scores of interoceptive accuracy, interoceptive confidence, interoceptive awareness, tactile acuity and phenomenological control were entered as mean-centred variables to the model predicting dis-ownership scores on the ‘long’ version of the mixed-reality task, along with the additional two-way interactions with the Delay and Source conditions. None of the additional predictors were individually significant.

These findings are identical to the multiple regression approach reported in the primary manuscript.

1. **Questionnaire distributions**

The total score of the Cambridge Depersonalisation Scale ranged from 0 to 118, with a mean of 36.18 (SD = 24.39). A Shapiro-Wilk test suggested that the data was non-normally distributed, $W=0.95$, $p<.001$.

The total score of the Cardiff Anomalous Perceptions Scale ranged from 0 to 28, with a mean of 6.25 (SD = 4.99). A Shapiro-Wilk test suggested that the data was non-normally distributed, $W=0.91$, $p<.001$. The subscales of distress, $W=0.84$, $p<.001$, intrusiveness, $W=0.87$, $p<.001$ and frequency, $W=0.79$, $p<.001$ were also non-normally distributed.

Histograms displaying the distributions of each questionnaire are presented in Figure S1.


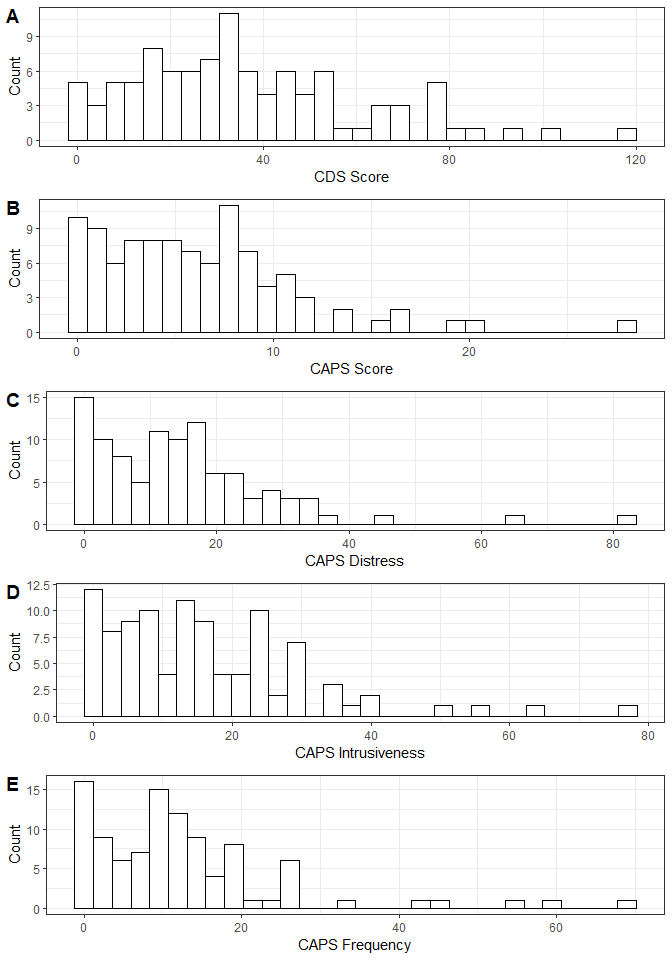


*Figure S2.* **Questionnaire Distributions.** Histogram displaying the distribution of total scores in response to the Cambridge Depersonalisation Scale (CDS) in graph A and the Cardiff Anomalous Perceptions Scale (CAPS) in graph B. Histograms for the distribution of CAPS Distress, Intrusiveness and Frequency are presented in graphs C, D and E respectively.

1. **Heartbeat Discrimination Task**
   1. **Division of Confidence into Quantiles**

In order to calculate meta-d’, confidence scores were first divided into four quantiles, meaning each confidence rating was binned according to where it fell in the distribution of all scores. Scores lower than 0.37 (25th Quantile) were labelled as 1, scores between 0.37 and 0.57 (50th Quantile) were labelled as 2, scores between 0.57 and 0.71 (75th Quantile) were labelled as 3 and scores between 0.71 and 1 were labelled as 4. The distribution of confidence scores and the division of confidence ratings into 4 bins are presented in Figure S2.


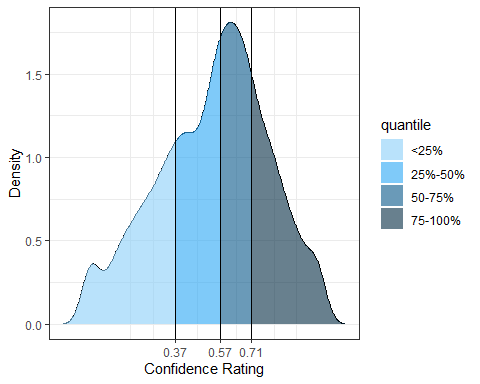


Figure S3. **Heartbeat Discrimination Confidence Bins.** Density plot demonstrating the division of confidence ratings into 4 bins based on quantile analysis of confidence ratings.
